# The magic mirror: a novel intraoperative monitoring method for parathyroid glands

**DOI:** 10.3389/fendo.2023.1160902

**Published:** 2023-05-22

**Authors:** Yue Yuan, Xiao Li, Xin Bao, Mingmei Huangfu, Hong Zhang

**Affiliations:** Department of Thyroid Surgery, The Second Hospital of Jilin University, Changchun, China

**Keywords:** parathyroid gland, near-infrared, autofluorescence, indocyanine green, thyroid surgery

## Abstract

The accurate detection of parathyroid glands (PGs) during surgery is of great significance in thyroidectomy and parathyroidectomy, which protects the function of normal PGs to prevent postoperative hypoparathyroidism and the thorough removal of parathyroid lesions. Existing conventional imaging techniques have certain limitations in the real-time exploration of PGs. In recent years, a new, real-time, and non-invasive imaging system known as the near-infrared autofluorescence (NIRAF) imaging system has been developed to detect PGs. Several studies have confirmed that this system has a high parathyroid recognition rate and can reduce the occurrence of transient hypoparathyroidism after surgery. The NIRAF imaging system, like a magic mirror, can monitor the PGs during surgery in real time, thus providing great support for surgeries. In addition, the NIRAF imaging system can evaluate the blood supply of PGs by utilizing indocyanine green (ICG) to guide surgical strategies. The NIRAF imaging system and ICG complement each other to protect normal parathyroid function and reduce postoperative complications. This article reviews the effectiveness of the NIRAF imaging system in thyroidectomies and parathyroidectomies and briefly discusses some existing problems and prospects for the future.

## 1 Introduction

The accurate identification of the parathyroid is essential in various types of thyroidectomies and parathyroidectomies (1). The adequate protection and effective removal of the parathyroid in thyroidectomy and parathyroidectomy impact patients’ prognosis (2). In thyroid surgery, accidental excision of PGs caused by the failure to identify them will result in hypoparathyroidism that has an impact on temporary or persistent hypocalcemia presenting as paresthesia, muscle cramps, and numbness (3). Oral calcium and calcitriol supplements may need to be taken for an extended period in cases of permanent hyperparathyroidism, which will not only increase the suffering and economic burden of patients but also directly affect their outcomes (2, 4). Moreover, for patients with a parathyroid disease requiring surgical treatment, if the diseased PGs are omitted, patients will continue to be affected by the illness and confronted with a second surgery (5, 6).

Since the development of neck surgery, researchers have developed various methods to identify PGs. Overall, experienced surgeons identify the PG by visual examination and palpation, which is considered the most common method (7, 8). Moreover, the invasive histopathological method is considered the gold standard for confirming PGs (9). Meanwhile, numerous non-invasive imaging methods are applied, including the ^99m^Tc sestamibi scan with sensitivity between 70% and 90%, ultrasound (70%–85%), and the relatively rarely used methods of 4D computerized tomography (CT) and magnetic resonance imaging (MRI) (10–15). These non-invasive imaging methods described above have long histories and some limitations, such as being preoperative only and non-real-time. Additionally, the use of methylene blue and aminolevulinic acid as intraoperative adjuncts can aid in the search for the PGs; however, the disadvantages of toxic metabolic encephalopathy (16, 17) and the need to shield patients from light for an additional 48 h after surgery make their application limited (18, 19).

**Figure 1:**
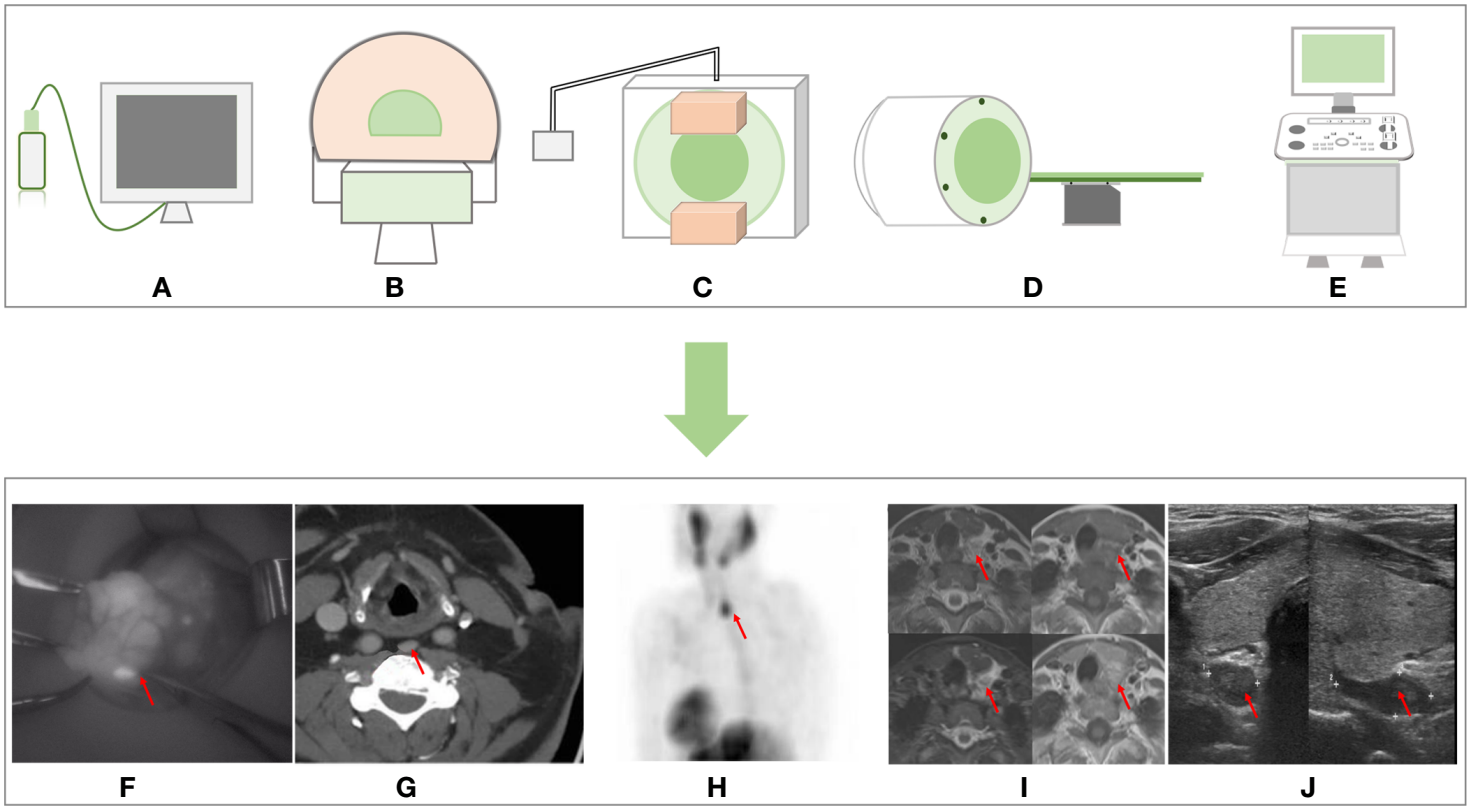
Non-invasive imaging methods sample graphs and the imaging effect pictures. **(A)** NIR imager. **(B)** CT. **(C)** ^99m^Tc sestamibi scan. **(D)** MRI. **(E)** Ultrasound. **(F)** NIR imaging. **(G)** CT imaging from Moska Hamidi et al. (14). **(H)** ^99m^Tc sestamibi scan imaging and **(J)** ultrasound imaging from Sharanya J Sanka et al. (22). **(I)** MRI from Seyma Yildiz et al. (13). NIR, near-infrared.

In view of this, novel assistive technology is advancing. In recent years, near-infrared (NIR) imaging and indocyanine green (ICG)-enhanced fluorescence have become highly debatable. Invasive examinations such as frozen section examinations have been widely used to confirm PGs (20); nevertheless, this non-invasive method benefits patients in terms of cost-effectiveness and accuracy rate of parathyroid identification (6). Thus, surgeons can improve visualization and be provided with detailed anatomical information during different surgical procedures to determine the next surgical strategy (21). We summarize the effect and significance of its application in thyroidectomy and parathyroidectomy in this literature and aim to offer help to guide the application of near-infrared autofluorescence (NIRAF) along with ICG (**Figure 1** and **Table 1**).

**Table 1:** The development of non-invasive imaging methods for detecting PGs.

Non-invasive imaging methods	Start time	Author	Reference
Ultrasound	1965	L Lacroix	(23)
Computed tomography	1977	J L Doppman	(24)
Magnetic resonance imaging	1984	D D Stark	(25)
^99m^Tc sestamibi scan	1989	A J Coakley	(26)
Near-infrared imaging	2011	Paras	(27)

PGs, parathyroid glands.

## 2 Description of parathyroid gland

### 2.1 Anatomy of parathyroid gland

**Figure 2:**
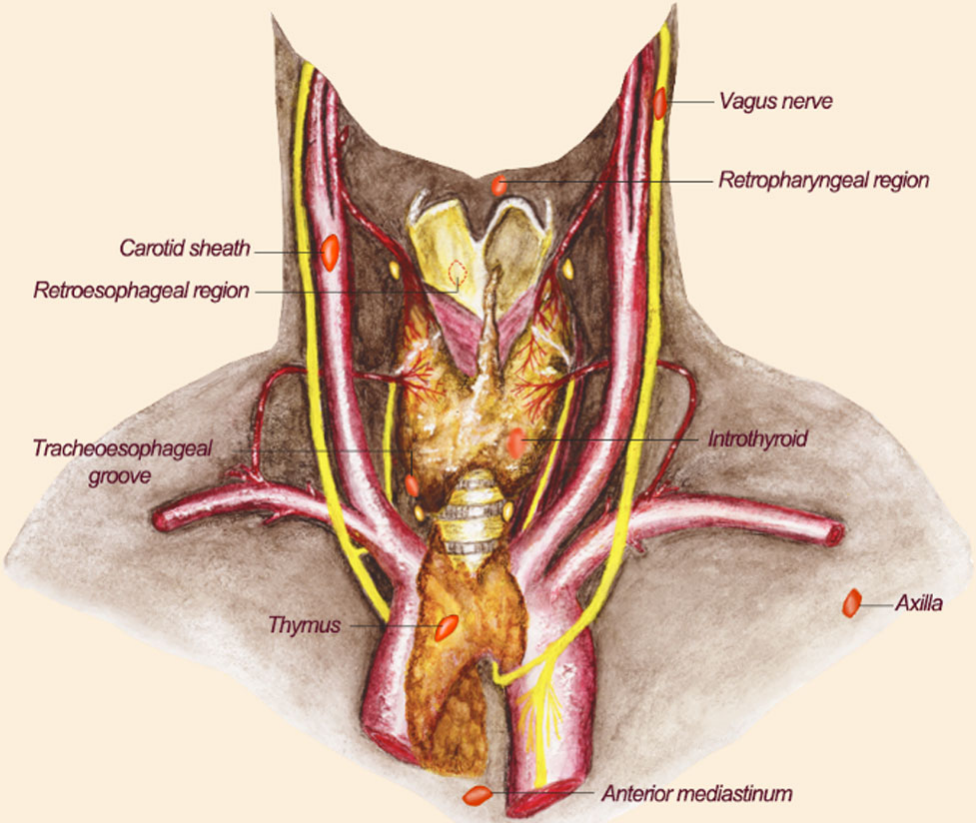
The location of ectopic parathyroid glands. The possible location distribution of ectopic PGs is shown here. The reddish-brown elliptic structures represent PGs. Their positions are indicated by text in the picture. PGs, parathyroid glands.

The PGs lie on the back of the left and right lobes of the thyroid glands, with colors varying from red to brown and being small in size (28). Four PGs account for 80% of the population (29). The PGs are located in the thyroid surgical capsule and encased in adipose tissues (30). The superior parathyroid gland (PG) is generally located abaxial to the superior lobe of the thyroid glands, with a lower probability of ectopic. However, the position of the inferior PG varies greatly, and it can appear near the inferior pole of the thyroid gland, in the central lymph nodes, or another distant location (28, 31–33). The rate of ectopic PGs attained is 35% due to abnormal migration during early development (34). The ectopic PGs may be present in the anterior mediastinum, tracheoesophageal groove or retroesophageal region, the retropharyngeal region, the axilla, along the course of the vagus nerves, near the carotid sheath, in the thymus, and within the thyroid gland (35). Furthermore, the tiny volume and the similar appearance to the adipose tissue and the lymph nodes lead to an increased difficulty for surgeons to identify (2). PGs in unconventional locations are difficult to identify in the field. In many cases of thyroid surgery, ectopic PGs in the operative field tend to be mistakenly severed or damaged as the adipose tissue, thus losing their function (36). Deliberate attempts to locate and protect all the operative PGs are not ideal. This may extend the time of operation and enlarge the wound surface. Similarly, the ectopic lesion of PGs increases the operative difficulty for the surgeon during parathyroidectomies. Although doctors usually utilize imaging to locate the PGs before surgery (37), the intraoperative visual picture is always more complex and challenging. Therefore, in thyroid surgery, simple and efficient auxiliary means to identify the PGs are highly desirable (**Figure 2** and **Table 2**).

**Table 2:** The location of ectopic parathyroid glands.

The location of ectopic PGs	The percentage of ectopic PGs	Reference
Mediastinum	13%	(38)
Thymus	7%	(39)
Within the thyroid gland	5%	(40)
Tracheoesophageal groove or retroesophageal region	1%	(40)
Retropharyngeal region	<1%	(30)
Carotid sheath	<1%	(41)
Vagus nerves	–	(42)
Axilla	–	(43)

PGs, parathyroid glands.

Further, the vascularization of PGs is fragile (44). Most of the superior and inferior PGs receive blood supplies from the inferior thyroid artery. Approximately 15% of superior PGs and 5% of inferior PGs are supplied by the superior thyroid artery. The remaining PGs may rely on the anastomoses between the two arteries (45). The blood vessels surrounding the PGs are very thin and fragile, resulting in an increased rate of devascularization. Thus, the protection of the vascularization of PGs is highly important to prevent secondary hypocalcemia caused by hypoparathyroidism (46).

### 2.2 Function of parathyroid gland

The PGs are endocrine organs that secrete parathyroid hormone (PTH). PTH acts on osteoclasts to bring bone calcium into the blood, increasing the blood calcium. Meanwhile, through the action of PGs, renal tubules reduce phosphorus reabsorption and blood phosphorus (47). Once the surgeon accidentally damages the PG during thyroidectomy, hypoparathyroidism with or without clinical manifestations will occur, which results in symptoms such as paresthesia, muscle cramps, and numbness associated with hypocalcemia (48–50). However, when patients suffer from primary or secondary hyperparathyroidism (HPT), surgeons always perform parathyroidectomy, including partial and total parathyroidectomy. In the course of both procedures, the leakage of diseased PGs in parathyroidectomy results in the recurrence of HPT and an increased risk of second surgery (51). Furthermore, we need to protect the function of PGs left by surgeons on purpose in parathyroidectomy to prevent postoperative hypoparathyroidism and reduce the burden and pain of the patients (4). Therefore, accurate protection of robust parathyroid function and radical resection of the diseased PG is necessary.

## 3 Near-infrared autofluorescence as the novel adjunct in surgery

### 3.1 Near-infrared fluorescence

The NIR fluorescence imaging system is a promising adjunct to modern surgery. Because of its non-invasive characteristics, high sensitivity, and instantaneity, the technique is now widely used in surgery (52). The NIR instruments launch the NIR light onto the tissue surface and differentiate between normal and diseased tissues through the inherent differences in the optical properties of various tissues (53). The NIR-guided imaging system has the advantages of low inherent autofluorescence (AF) background and real-time imaging (54). Furthermore, enhancing the optical contrast between normal and diseased tissues can aid in diagnosis and treatment. Because most tissues generate little NIR fluorescence, most *in vivo* studies administer exogenous contrast agents to attain the goal of directing surgery (55).

In diagnosing and treating many diseases, some common imaging methods applied intraoperatively cannot be employed as real-time tools. Although intraoperative MRI and CT scanning have equally played a significant role (56), intraoperative systems are costly and complex, thus limiting their use (57). As a novel technique, NIR fills the gap between preoperative imaging and intraoperative reality (58). Specialized intraoperative NIR imaging systems for open surgery (59), laparoscopy (60), thoracoscopy (61), and robotic surgery (62) enable surgeons to achieve real-time guidance during surgery (57). The most widely used applications include visualization of tumor incisal margins with fluorescent dyes, identification of sentinel lymph nodes, and evaluation of tissue perfusion (63, 64). Currently, NIRAF is constantly evolving and seeking new fields of interest.

### 3.2 Autofluorescence

Autofluorescence is located on the endogenous fluorophores in tissues and requires radiation of a suitable wavelength to be distinguished from fluorescent signals obtained by adding exogenous markers (54). It is used in many fields, such as ocular fundus pathology (65), the localization of parathyroid lands (66), AF pleuroscopy (67), and the identification of tumors (52, 68). Paras et al. first discovered the AF potential of PGs by using a fiber optic spectrometer to compare the fluorescence intensity of different neck tissues exposed to near-infrared light (27). Since then, parathyroid imaging has advanced rapidly.

PGs demonstrate greater fluorescence intensity than thyroid fluorescence without the need for exogenous chemicals excited by NIR fluorescence (27). At present, various mechanisms of parathyroid AF have been mentioned in different literature; however, no accurate conclusion has been reached. A relatively popular explanation is that the calcium-sensing receptor (CaSR) causes parathyroid AF due to its high expression in PGs. Contrastingly, thyroid tissue expresses CaSR at a lower level, similar to the muscle, fat, and lymph of the neck region (69). Furthermore, lipofuscin, present in the active chief cells of the PG, is also an important factor. It accumulates gradually in the human body with age, which may explain the higher fluorescence intensity of PGs in adults than in children under 12 years old (70). Other potential fluorophore candidates include oxyphil predominance (71), secretory granules, and porphyrin derivatives (72). Although the exact mechanism of action remains unclear, parathyroid AF combined with the NIR imaging system still has a high clinical value (73, 74).

### 3.3 NIRAF imaging system in surgery

The NIRAF imaging system can achieve imaging by AF from human tissues without additional reagents. Therefore, NIRAF-guided surgery is widely applied as an emerging auxiliary means to help surgeons observe the location and anatomy of autofluorescent tissues (75). In the ophthalmology department, fundus AF can be measured non-invasively by NIRAF, providing a diagnostic message for macular diseases (76). NIRAF helps to identify retinal laser injury (77) and uses melanin AF to detect early retinal pigment epithelium alterations and diagnose choroidal melanoma malignancy (78–80). The NIRAF imaging system is occasionally used to detect high-risk plaques in patients with coronary artery atherosclerosis. Some researchers found a significant association between NIRAF and intraplaque hemorrhage and ceroids in high-risk plaques (81, 82). Moreover, studies have shown that other endocrine organs or related diseases, such as the adrenal (83) and neuroendocrine tumors (84), can also produce AF.

### 3.4 Application of the NIRAF imaging system for the identification of parathyroid glands

**Figure 3:**
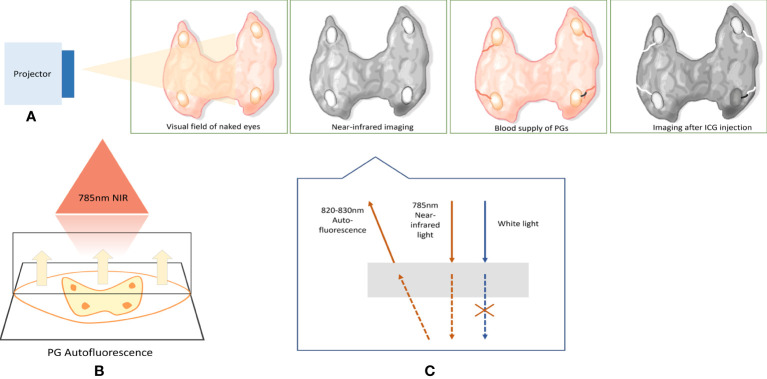
Working diagram of NIRAF imaging system. **(A)** Simulates the effect of NIR imaging device irradiation on the surgical field and shows the ICG angiography. When the NIR imaging system illuminates the surgical field, the PGs will emit the AF along with the black setting from other tissues. After intravenous injection of ICG, the blood vessels of PGs will appear. The black lines indicate the devascularization of the PG; thus, the PG, whose blood supply is damaged, will eventually darken in contrast with the high- brightness background. **(B, C)** The principle of NIR imaging system. The projector emits 785-nm NIR light. Then, the PGs emit AF with 820–830-nm wavelength. NIRAF, near-infrared autofluorescence; ICG, indocyanine green; PGs, parathyroid glands.

NIRAF can distinguish the imaging between parathyroid and adjacent tissues, including the adipose tissue, lymph nodes, and thyroid glands. It can also discern the localization of the PGs in the operative field in real-time (6, 27). The application process of the NIRAF imaging system is similar to most of the studies previously summarized. During the operation, surgeons need to turn off the room’s operating lights and use the projector to irradiate the operative field (85). PGs emit AF when stimulated by a 785- nm wavelength laser, with peak fluorescence occurring at 820–830 nm (27). The AF is derived from high concentrations of intrinsic NIR fluorophores in the PG. Except for parathyroid carcinoma, which is currently controversial, PGs produce AF in most physiological and pathological conditions, whose fluorescence is 2.4 to 8.5 times brighter than that of surrounding tissues (72); glowing PGs can then be observed on the screen with a black setting from other tissues. The whole process only takes approximately 30–40 s to identify PGs without damaging the tissues (69). The parathyroid AF remains stable over time, even lasting for at least 1 h after resection (86). In addition, NIRAF parathyroid observation revealed the AF intensity threshold at optimal normalization, which was 1.72 on the receiver operating characteristic (ROC) curve. This study indicated that the utility of AF thresholds for the highest specificity of parathyroid identification could avoid 40% of frozen sections to confirm parathyroid tissue (87) (**Figure 3**).

So far, various devices have been put into clinical use based on the principle of NIR irradiation to trigger parathyroid AF. The PDE-NEO (88) and Fluobeam series, including the FLUOBEAM^®^ LX (89) and Fluobeam 800 (86), can detect PGs in open neck surgery and can be operated on in conjunction with ICG. In addition, the PTeye system (90), in the form of a probe, can also detect PGs independently or in combination with ICG. Others, like PINPOINT^®^ + SPY-PHI, IMAGE S™ RUBINA, and EleVision™ IR Platform, can be used in conjunction with ICG under open surgery and laparoscopy (91). As for the Quest Spectrum^®^ (92), it can only be used in combination with ICG under open surgery, which does not have the function of self-fluorescence detection.

The NIRAF imaging system demonstrated high accuracy in almost all studies. Paras et al. were the first to demonstrate the feasibility of parathyroid imaging using the NIRAF principle. Real-time parathyroid exploration was performed in 21 patients. All the PGs showed greater fluorescence intensity than the surrounding tissues (27). The data obtained are impressive in a decade of clinical studies of the NIRAF imaging system. The highest parathyroid detection rate was 100% (93, 94), the lowest was 78.64% (95), and the rest were over 90% (96, 97). Studies found that certain thicknesses of tissues obscuring the PGs and the pre-anatomical application of the system became the major influencing factors in the inability to detect PGs (66, 98). In another instance, the use of the system has led to an obvious decrease in the rate of inadvertent PG resection in all studies where statistical data are available (99, 100). The same was true for the auto-transplantation rate of PGs (99, 101).

Based on the feature of accurate identification of PGs using the NIRAF imaging system, several studies have analyzed its effect on protecting parathyroid function by comparing postoperative blood calcium and parathyroid levels in patients with and without the NIRAF system. In most clinical trials, the application of the NIRAF imaging system can reduce temporary postoperative hypocalcemia and hypoparathyroidism rates (96, 99, 101, 102). A meta-analysis collected six articles that included 2,180 patients. The final result showed that the temporary postoperative hypocalcemia rates were 25.19% in the control group and 8.11% in the NIRAF group (103). Interestingly, for permanent postoperative hypocalcemia and hypoparathyroidism, almost all the relevant research implied that this technique was not capable of improving the long-term outcomes of patients (96, 99, 103). In existing randomized controlled studies, researchers have focused on the comparison between postoperative hypocalcemia and hypoparathyroidism. Most studies have shown that using the NIRAF system can improve the parathyroid identification rate and reduce the incidence of postoperative transient hypocalcemia and hypoparathyroidism; however, it cannot reduce the incidence of permanent hypoparathyroidism. Due to the small sample size and the single-blinded study design, some studies showed no significant difference in the probability of temporary hypocalcemia between the two groups (96, 100, 101). Overall, the group using the NIRAF system had lower rates of hypocalcemia and hypoparathyroidism. Furthermore, a team studied the influence of a single intraoperative application of the NIRAF imaging system on postoperative parathyroid insufficiency after thyroidectomy. The research finding shows that only continuous use of the NIRAF imaging system might enhance treatment outcomes (89) (**Table 3**).

**Table 3:** The summary of NIRAF application in identification of PGs.

Year	Number of patients	The rate of identification of PGs	The rate of inadvertent PGs resection (control group *vs.* NIRAF group)	Auto-transplantation rate (control group *vs.* NIRAF group)	Temporary postoperative hypocalcemia rate (control group *vs.* NIRAF group)	Permanent hypocalcemia rate (control group *vs.* NIRAF group)	Temporary hypoparathyroidism rate (control group *vs.* NIRAF group)	Permanent hypoparathyroidism rate (control group *vs.* NIRAF group)	Ref.
2022	76	78.64%	N/A	N/A	N/A	N/A	N/A	N/A	(95)
2022	60	N/A	N/A	37% *vs.* 27%	N/A	N/A	33% *vs.* 20%	N/A	(101)
2021	180	N/A	28.9% *vs.* 14.4%	N/A	5.6% *vs.* 3.2%	N/A	25.9% *vs.* 27.7%	N/A	(100)
2021	542	93.50%	12.8% *vs.* 6.9%	10 PGs (no rate)	2.5% *vs.* 2.3% (no significant difference)	1.1% *vs.* 1.1% (no significant difference)	18.9% *vs.* 8.8%	4.6% *vs.* 4.2% (no significant difference)	(96)
2021	2180	N/A	N/A	N/A	25.19% *vs.* 8.11%	2.19% *vs.* 0% (no significant difference)	N/A	N/A	(103)
2020	241	N/A	11.7% *vs.* 2.5%	13.3% *vs.* 3.3%	21.7% *vs.* 9.1%	1.6% *vs.* 0% ((no significant difference)	N/A	N/A	(99)
2018	162	92.50%	N/A	N/A	N/A	N/A	N/A	N/A	(90)
2016	137	97%	N/A	N/A	N/A	N/A	N/A	N/A	(97)

NIRAF, near-infrared autofluorescence; PGs, parathyroid glands.

Different states of PGs may show different images under the irradiation of the NIRAF imaging system. Parathyroid adenomas demonstrated a heterogeneous NIRAF pattern, with adenoma tissues documented as having significantly less AF than the rim of normal parathyroid tissues in some research (98, 104, 105). However, not all studies show this difference. The relevant studies showed high-intensity fluorescence in PGs with different states (90, 106, 107). The results of different pathological types of PGs were confusing and inconclusive. In patients with secondary HPT and parathyroid adenoma, the NIRAF imaging system fails to make a clear distinction (98). On the contrary, research indicated that the AF intensities of primary HPT were stronger than those of secondary HPT (88). In summary, regardless of the state and pathological type of the PGs, the NIRAF imaging system can distinguish them well from the surrounding tissues, even though discrepancies may exist in the images.

In the process of NIRAF imaging, the factors influencing parathyroid AF intensity may include the following: McWade et al. proposed that well-differentiated papillary carcinoma may reduce parathyroid AF intensity (97). However, no scholars have conducted subsequent specialized research on this aspect. A previous study indicated that the AF intensity of PGs may correlate negatively with PTH concentration (71). The weight of PGs and patients’ age may impact the primary HPT AF intensity (108). Interestingly, the number of PGs found in the NIRAF imaging system is significantly higher than in white light. This difference is three times more likely to be found in patients with thyroiditis than in those without (109).

Based on these studies, we conclude that NIRAF has a satisfactory parathyroid identification rate, which makes it a reliable auxiliary equipment (110). In thyroid surgery, NIRAF is also significantly effective in reducing transient hypocalcemia after surgery. In parathyroid surgery, although it is impossible to distinguish pathological types of PGs, the ability to identify and locate PGs is still worthy of recognition. Moreover, there are few studies on the influencing factors of parathyroid AF, and the results remain inconclusive.

## 4 The partner of NIRAF: indocyanine green

### 4.1 Indocyanine green

ICG, with a molecular weight of 774.96 Da, is a water-soluble anionic amphiphilic tricarbocyanine dye (111). It can be injected into the human bloodstream or indirectly into lymphatic vessels through injection into tumors, and it gives off fluorescence through excitation by the NIR spectrum (21, 112). The maximum absorption spectrum is 805 nm, and re-emission is 835 nm (113). ICG binds tightly to plasma protein and circulates in the intravascular compartment or lymphatic vessel, making it possible to visualize perfusion with a NIR camera (21, 45). The liver takes up ICG and rapidly excretes it into the bile without undergoing biotransformation or enterohepatic circulation (114) with practically no adverse effects (115).

ICG, authorized by the Food and Drug Administration, has become a safe and cost-effective dye with a half-life of 3–5 min (58) and is eliminated from the liver 15–20 min after injection. ICG is not a selective fluorescence agent that can be used for a specific organ. However, organs that have more blood supply show stronger fluorescence than surrounding tissues, and this process helps define the organs’ or tissues’ borders (116). Nevertheless, ICG also has some disadvantages, including limited photostability, a moderate fluorescence quantum yield, a high plasma protein binding rate, and undesired aggregation in an aqueous solution (64). In conclusion, ICG is still a reliable and promising contrast agent worthy of use in surgery.

### 4.2 Indocyanine green in surgery

ICG has been widely used in surgery because of its molecular properties and characteristics. It always serves as a contrast agent in the blood vessel or the body’s plumbing. The surgeons use intraoperative ICG fluorescence imaging to explore the hepatobiliary anatomy, outline the ureter enteric stricture margins, identify the structured ureter and urinary diversion, and so on (117, 118). In addition, the use of ICG is well- established for checking anastomotic stump perfusion in visceral surgery (119). In urology, ICG is applied for vasculature identification and assessment of allograft perfusion in nephrectomy and kidney transplantation (117). In general surgery, ICG plays an important role in assessing anastomosis blood perfusion in colorectal surgery and evaluating the perfusion of the colonic stump, which may reduce the risk of anastomotic leaks (120). Meanwhile, in plastic surgery, ICG angiography is used to assess free flap anastomosis and design skin paddles (121). In surgery, ICG has become an available adjunct for identifying malignant tumors and tracking metastases. A study reviewed the applications of ICG in brain tumor surgery and found it useful for intraoperative visualization of the most common brain tumors and localizing intraparenchymal metastases (122). A similar impact is also demonstrated in hepatocellular carcinoma, hepatoblastoma, colorectal tumors, and genitourinary cancer (123). Moreover, using ICG is a feasible and seemingly reliable method for lymphatic mapping in varieties of cancers like breast cancer, endometrial cancer, and cervical cancer (124, 125).

## 5 ICG-NIR fluorescence imaging

### 5.1 The use of ICG-NIR fluorescence imaging in different surgical procedures for parathyroid protection

The NIR fluorescence imaging system and ICG intraoperative administration are widely used in various types of thyroidectomies and parathyroidectomies, which refer to traditional thyroidectomy, robotic thyroidectomy, endoscopic thyroidectomy, traditional parathyroidectomy, and video-assisted parathyroidectomy (2, 8, 126). The application processes are all very similar. Due to the depth of NIR, radiation only reaches a depth of approximately 10 mm (75). Surgeons need to illuminate the operative field with a NIRAF projector when the thyroid gland is retracted (106, 127). The adipose tissue around the PG may influence the effect and cause a negative result (27). The application of the NIRAF imaging system can realize the role of position. The intravenous administration of ICG is as follows: in endoscopic surgery, some scholars choose to inject ICG 1 h before anesthesia (127). Currently, most surgeons prefer to administer the drug intravenously after thyroid exposure, despite the technique used (128, 129). During surgery, multiple doses can be administered according to real-time requirements, usually 5–20 mg each time, and the total injection dose should not exceed 2 mg/kg (130). In some cases, a single assessment of the preserved parathyroid blood supply after thyroidectomy has been performed, and if the blood supply is lost, auto-transplantation has been selected (131). The imaging results after the ICG injection were satisfactory. The glands can be visually identified in approximately 30–60 s after administration (129). Assessing the vascularization of PGs is implemented by using ICG. The slight heterogeneity of the surgery process may exist among different operators. When devascularization occurs, parathyroid auto-transplantation is required (132). The whole process aims to protect the normal PGs in thyroidectomy and the remnant PGs in parathyroidectomy to prevent hypoparathyroidism (126).

### 5.2 ICG-NIR fluorescence imaging in thyroidectomy and parathyroidectomy

The NIRAF imaging system demonstrates an excellent ability to identify and locate PGs. However, the protection of PGs is not equal to the protection of parathyroid function. It is essential to preserve the feeding vessels and guarantee the perfusion of PGs to prevent postoperative hypoparathyroidism (94). Compared to using the NIRAF imaging system alone, ICG-NIR fluorescence imaging can identify PGs and evaluate blood supply. We have summarized the related research in recent years and presented the results and data in this review.

It has been reported in many studies that the ICG-NIR imaging fluorescence also has considerable parathyroid recognition ability (131, 133, 134). A comparison of the sensitivity of identifying PGs was made between NIRAF and ICG imaging. The results were 82% and 81%, respectively, with no significant difference (134). Similar results were found in Kahramangil and Berber’s study, in which there was no significant difference in the parathyroid detection rate of AF and ICG, and both performed well (135). Additionally, Iritani et al. proposed that central neck dissection and lateral neck dissection were significantly associated with postoperative hypoparathyroidism. The position is adjacent to the central lymph nodes, and the malignant pathological type improves the risk of inadvertent resection (136). Interestingly, ICG-NIR fluorescence imaging becomes more available with repeated surgery and the discrimination of PGs from the lymph nodes in cases of thyroid malignancy (128).

In unconventional thyroidectomies and parathyroidectomies, the specialized endoscope ICG-NIR imaging system is designed to explore PGs. Although the endoscope has its advantage in identifying PGs through the magnification of the operative field, many cases barely visible to the naked eye still rely on the ICG-NIR imaging system (2). Some scholars have applied the ICG-NIR imaging system to bilateral axillo-breast approach robotic thyroidectomy. In their study, the probability of incidental parathyroid resection was significantly lower in the ICG group than that in the control group. However, the postoperative hypocalcemia and hypoparathyroidism rates show no significant difference between the groups (126). A previous study shows that the technique is also suitable for video-assisted neck surgery (2). Unfortunately, perhaps due to the small sample size in endoscope ICG-NIR imaging studies, no significant difference was observed in postoperative complications.

### 5.3 Scoring system for detecting parathyroid by ICG-NIR fluorescence imaging system

In general, the system can be used to assess parathyroid blood transport by ICG imaging. However, there has been no uniform standard to classify the intensity of parathyroid imaging after the injection of ICG or to evaluate under what fluorescence intensity the parathyroid perfusion is good. Through research in recent years, we have found three main scoring methods. In a 2016 study, the team formulated a grading scale based on the area of fluorescence in the PGs (12). 1+, 2+, and 3+ represent uptake in <30% , 30–70%, and >70% of the gland, respectively. ICG fluorescence is present in all the PGs. The degree of uptake is 1+ in 13.4%, 2+ in 22.3%, and 3+ in 57.1%. Postoperative hypoparathyroidism was not followed up. Moreover, the research suggests that the imaging method is of limited use in patients undergoing first-time neck surgery because of the impact of thyroid fluorescence. Another scoring criterion is based on the fluorescence brightness of the PGs compared to the fluorescence brightness of the surrounding tissues (137). The fluorescence intensity score includes a 1–3 grading scale, which means no visible fluorescence, fluorescence exists but no more than the surrounding tissues, and parathyroid is more fluorescent than the surrounding tissues. The higher the score, the lower the rate of postoperative hypoparathyroidism. Lastly, intraoperative mapping angiograms of the PG (iMAP) score are divided into three levels, iMAP 0, iMAP 1, and iMAP 2, representing the perfusion of PGs that varies from no information to a clear vascular pedicle flowing into the PG (45, 138). iMAP can offer vascular information directly in one-third of cases and needs to improve. The research also provides no information on postoperative complications (138). The above three systems are ineffective; therefore, further study and induction are needed to develop a unified and authoritative standard for evaluating the fluorescence intensity of the PGs.

According to the research, ICG-NIR fluorescence imaging contributes to thyroidectomy and parathyroidectomy. However, the problems, including the small sample size and incompleteness of research in some studies, obscure the imaging method’s advantages. The technique has huge potential, and future research is still worthwhile.

## 6 Discussion

In the last 10 years, research has been conducted on the intraoperative identification and protection of PGs using NIRAF imaging and ICG-NIR fluorescence imaging methods simultaneously. As for the capacity of parathyroid identification, various studies have obtained relatively consistent results, i.e., high precision and accuracy (90, 97). However, intraoperative use of the NIRAF imaging system and ICG-NIR fluorescence imaging produced good results due to multiple confounding factors. For example, in the same study, the surgical methods and diseases are not uniform, the data collection is not comprehensive, and the sample size is small; therefore, there is considerable heterogeneity in some studies. Furthermore, we found that the ability of the system to prevent temporary hypoparathyroidism and hypocalcemia after surgery was ambiguous in all studies (96, 100). In a unilateral or partial thyroidectomy, since the intact PG is present, postoperative blood calcium levels and PTH levels can be compensated by the normal PG, resulting in no significant difference in laboratory results with or without this technique. The measurement of postoperative blood calcium and PTH levels is significant for bilateral thyroidectomy or surgery with bilateral thyroid exploration. The application of the system does not affect the occurrence and development of permanent postoperative hypoparathyroidism and hypocalcemia (96, 103). However, it still aids surgeons in making sound decisions.

The intensity and pattern of parathyroid AF in different states and pathological types vary in relevant research (104). At present, there is no uniform conclusion on the fluorescence pattern of different pathological types of PGs, which may be related to different models of NIRAF devices or patient factors. In general, such heterogeneity does not affect the recognition rate of PGs. Although different pathological types of PGs have different imaging styles under near-infrared light, the differences between PGs and surrounding tissues are still obvious in most studies. We believe it is impossible to distinguish the pathological types and health status of PGs with the NIRAF imaging system. In addition, there are few studies on the influencing factors of parathyroid AF, and opinions vary. At present, these factors do not significantly affect the observation of intraoperative PGs.

The single application of NIRAF has played a significant role in the protection of PGs. However, the priority of parathyroid protection is to preserve blood transport. NIRAF alone cannot observe the blood flow of the PGs, so researchers applied ICG, a relatively mature angiography agent, to the technique to observe blood flow (12, 106). Even if ICG is applied before or during surgery, both methods can look for the blood flow of PGs successfully. Due to the rapid metabolism of ICG, most studies choose to inject ICG intravenously in real-time during surgery, and ICG can be applied repeatedly during surgery to achieve a continuous observation effect. In various studies, the ICG application dose varies from 2.5 to 20 mg (2, 126). The most appropriate ICG dose for the optimal visualization of PGs and background differentiation requires proof and research. Since ICG is injected intravenously into the body, it will remain wherever there is a blood supply; therefore, the dosage of ICG should be adjusted according to the actual needs. Excessive dosage may lead to a too-bright background and affect the observation of PGs. In addition, the standard for evaluation of parathyroid imaging is diverse in different studies (12, 137, 138). After ICG injection, there is no unified evaluation scheme for parathyroid perfusion that can guide clinical application. The lightening of the peripheral blood vessels of the PG confirms the good blood flow of the PGs, and the blackening confirms the loss of blood flow. However, the lower limit of the brightness—that is, the level of brightness that proves that the parathyroid function has been lost—is still unclear. In general, although there are still many aspects of this technique that are not unified and standardized, ICG combined with NIR has played a positive role in observing the state of the parathyroid.

In addition, the operative ability and experience of the surgeon are also notable influencing factors in this type of research. The ability of an experienced thyroid or endocrine surgeon to identify and protect the PGs remains superior even without the aid of the NIRAF system and ICG. However, the rate of intraoperative parathyroid recognition may be lower in younger, less experienced, or non-specialist physicians, such as those in ENT or general surgery (139). Similarly, the amount of thyroid and parathyroid surgery performed at a medical center also influenced the study. The larger the surgery volume and the more experienced the surgeon, the smaller the differences that occur between the NIRAF and control groups in the rate of parathyroid identification, postoperative hypocalcemia, and the incidence of hypoparathyroidism. Thus, the system benefits less experienced, younger physicians and physicians who are not thyroid or endocrine specialists.

Because of the advantages and the existing problems of the novel imaging method, opportunities and challenges exist simultaneously for researchers. This technique has practical clinical significance and promising development prospects but still needs improvement and perfection.

## 7 Conclusion

The NIRAF imaging system is a real-time imaging tool for detecting PGs that utilizes the intrinsic AF of the PGs to distinguish them from other tissues. Under appropriate anatomy and certain lighting conditions, the system can fully exploit the advantages of the specific identification of PGs. With high accuracy, sensitivity, and specificity, the system can locate PGs, thereby reducing the rate of inadvertent resection of healthy glands and eradicating diseased ones. ICG-assisted NIRAF imaging visualizes the vascularization of PGs and makes it possible to protect the blood supply and perfusion of the PGs. The novel intraoperative auxiliary imaging method can prevent the postoperative complications of temporary hypoparathyroidism and hypocalcemia from improving the prognosis of patients. Based on the advantages of this imaging method and the fact there are almost no adverse reactions, the NIRAF imaging system and ICG-NIR fluorescence imaging are expected to be techniques for conventional applications.

## Author contributions

YY have drafted the article. XL have revised the manuscript. XB and MH have contributed to the acquisition and interpretation of data. HZ has designed the work. All authors have contributed to manuscript revision, read and approved the submitted version.
